# The permeation mechanism of organic cations through a CNG mimic channel

**DOI:** 10.1371/journal.pcbi.1006295

**Published:** 2018-08-02

**Authors:** Luisa M. R. Napolitano, Arin Marchesi, Alex Rodriguez, Matteo De March, Silvia Onesti, Alessandro Laio, Vincent Torre

**Affiliations:** 1 International School for Advanced Studies (SISSA), Trieste, Italy; 2 Structural Biology Laboratory, Elettra-Sincrotrone Trieste S.C.p.A., Basovizza, Trieste, Italy; 3 INSERM U1006, Aix-Marseille Université, Parc Scientifique et Technologique de Luminy, Marseille, France; 4 ICTP, International Centre for Theoretical Physics, Trieste, Italy; 5 Cixi Institute of Biomedical Engineering (CNITECH), Nigbo Institute of Materials Technology and Engineering, Chinese Academy of Sciences, Zhejiang, P.R. China; 6 Center of Systems Medicine, Chinese Academy of Medical Sciences, Suzhou Institute of Systems Medicine, Suzhou Industrial Park, Suzhou, Jiangsu, P.R. China; Icahn School of Medicine at Mount Sinai, UNITED STATES

## Abstract

Several channels, ranging from TRP receptors to Gap junctions, allow the exchange of small organic solute across cell membrane. However, very little is known about the molecular mechanism of their permeation. Cyclic Nucleotide Gated (CNG) channels, despite their homology with K^+^ channels and in contrast with them, allow the passage of larger methylated and ethylated ammonium ions like dimethylammonium (DMA) and ethylammonium (EA). We combined electrophysiology and molecular dynamics simulations to examine how DMA interacts with the pore and permeates through it. Due to the presence of hydrophobic groups, DMA enters easily in the channel and, unlike the alkali cations, does not need to cross any barrier. We also show that while the crystal structure is consistent with the presence of a single DMA ion at full occupancy, the channel is able to conduct a sizable current of DMA ions only when two ions are present inside the channel. Moreover, the second DMA ion dramatically changes the free energy landscape, destabilizing the crystallographic binding site and lowering by almost 25 kJ/mol the binding affinity between DMA and the channel. Based on the results of the simulation the experimental electron density maps can be re-interpreted with the presence of a second ion at lower occupancy. In this mechanism the flexibility of the channel plays a key role, extending the classical multi-ion permeation paradigm in which conductance is enhanced by the plain interaction between the ions.

## Author summary

Cyclic Nucleotide Gated (CNG) channels are nonselective cation channels with a key role in sensory transduction. Despite sharing a high homology with K^+^ channels, they allow the passage of large compounds like dimethylammonium (DMA) which are not permeable through K^+^ channels. We demonstrate that the conduction mechanism of this compound is radically different from the textbook scenario, in which an ion, in order to diffuse through the channel, must cross a series of barriers, whose height is possibly perturbed by the presence of other ions in the channel. We show that permeation of large cations in CNG is due to the destabilization of the pore induced by the simultaneous presence of two ions in the channel.

## Introduction

Cyclic Nucleotide Gated (CNG) channels are nonselective cation channels opened by the direct binding of cyclic nucleotides, cAMP and cGMP. They play a key role in olfactory and visual signal transduction, generating the electrical responses to lights in photoreceptors and to odorants in olfactory receptors [1]. CNG channels are members of the voltage-gated ion channel (VGIC) superfamily that includes voltage-gated potassium (K_v_), sodium (Na_v_) and calcium (Ca_v_) and the transient receptor potential (TRP) channels [1,2]. They are heterotetramers composed of a combination of A subunits (CNGA1-CNGA5) and B subunits (CNGB1 and CNGB3) and, like all other members of the VGIC superfamily, each subunit contains six transmembrane α-helices (S1-S6) including a pore loop between S5 and S6 that forms the ion selectivity filter. Despite a significant homology with the highly selective K^+^ channels, CNG channels from both rod and cone photoreceptors do not discriminate among monovalent alkali cations and are permeable also to larger methylated and ethylated ammonium ions including dimethylammonium (DMA) and ethylammonium (EA) [3,4].

We have previously demonstrated that the filter of a CNG-like channel, named “NaK2CNG” channel, is rather flexible and dynamic [5]. However, an important—and at the moment unanswered—question is whether the permeation of large organic cations (i.e. the DMA) follows the same physical mechanisms of the alkali cations’ (i.e. K^+^ or Na^+^) permeation. Indeed, thermodynamic considerations and the results of Molecular Dynamics (MD) simulations have elucidated the mechanism of permeation of K^+^ and Na^+^ ions through ionic channels [6–12], demonstrating that the crossing of one or a few free energy barriers is the key limiting factor. In particular, at the selectivity filter a permeating ion, strongly hydrated in the bulk solution, has to lose some water from its hydration shell [8,13,14]. The free energy cost for dehydration is only partially compensated by the interactions gained in the binding site. Selectivity for K^+^ over Na^+^ arises when the difference in free energies of those ions in the pore departs from the corresponding difference in bulk solution [15]. In the multi-ion models, ions influence each other leading to the well-known anomalous mole fraction effect where the higher affinity ions effectively block the conduction of lower-affinity ions [6,16–18]. Indeed, Hodgkin and Keynes in their seminal paper [19] showed that the K^+^ channels can be occupied by more than one ion at a time, and ions hop in single file into vacant file with rate constants which depend on barrier heights, membrane potential and ion-ion repulsion. Recently, it has been proposed that destroying the multi-ion mechanism could lead to the nonselective ion conduction observed in the CNG channels [20,21] suggesting that the nonselective channels have a broken multi-ion mechanism [22,23].

**Fig 1:**
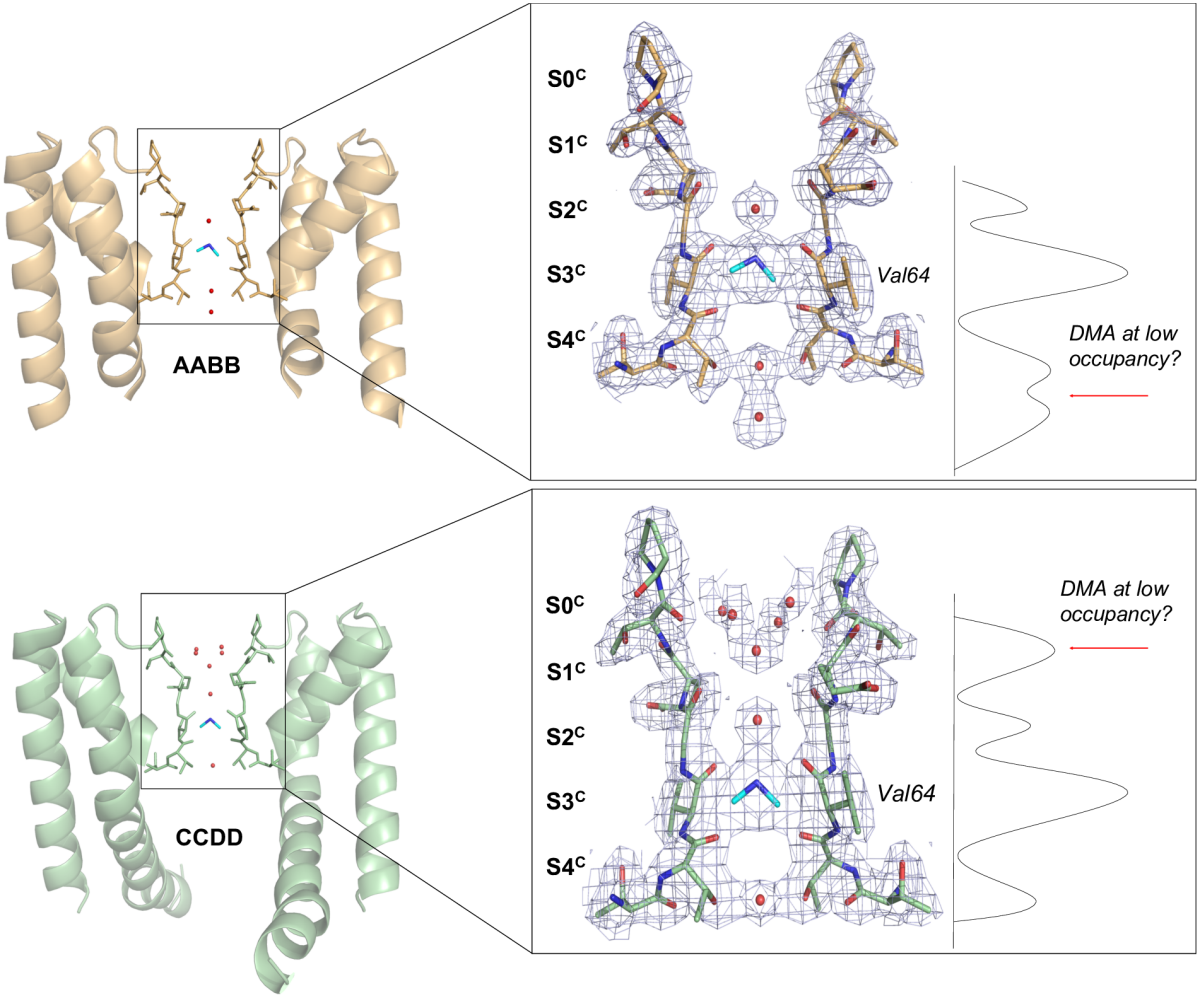
Crystal structure of the CNG-mimic:DMA complex. Overview of the AABB and CCDD NaK2CNG tetrameric structures (PDB ID: 4R7C) showing only two monomers for clarity; residues in the selectivity filter are shown as sticks. The black box indicates the region expanded in the right panel, corresponding to the selectivity filter. On the right of each molecule, the one-dimensional electron density profile along the axis of symmetry is reported. The DMA cation is shown in cyan and well-ordered water molecules are shown as red spheres. The crystallographic sites are labeled **S0-4**^**c**^, starting from the extracellular side. The final refined 2Fo-Fc electron density maps are shown at 1.3σ in light blue mesh. The position of Val64 is indicated.

While the permeation of Na^+^ and K^+^ ions and the mechanism for K^+^ selectivity has been widely studied, little is known about the permeation of larger molecules in ion channel. To address this point, we considered a NaK2CNG chimera channel, where the CNG selectivity filter (ETPP) was engineered onto a bacterial NaK channel. The NaK2CNG chimera, which extensive electrophysiological and crystallographic experiments have demonstrated to be a good model for the channel core [5,24,25], was crystallized in complex with DMA (PDB ID: 4R7C) (Fig 1) [5]. By combining electrophysiology and MD simulation within the Bias Exchange-Metadynamics (BE-META) scheme [5], we demonstrate that the permeation of DMA through the CNGA1 channels takes place by a different mechanism from the one governing the permeation of alkali cations [8,11,13,14,26]. Since DMA has two hydrophobic groups (CH_3_) which interact favorably with the hydrophobic groups present inside the channel pore, despite its large size, the organic cation enters into the channel core more easily than K^+^ or Na^+^ ions, forming a stable complex with a binding affinity of almost 50 kj/mol. We further show that the simultaneous presence of two DMA ions inside the channel significantly changes the pore structure, destabilizing the binding site that is observed with only one DMA. Importantly, we show that the rather significant conformational change induced by the presence of a second DMA is the key factor for the permeation. We thus propose that some organic molecules might permeate through channels by a mechanism in which the flexibility of the channel plays a key role, extending the single file hopping paradigm previously proposed (19–22).

## Results and discussion

### The free energy landscape of DMA through the CNG mimic pore

We started our analysis considering the crystal structure of the NaK2CNG chimera (23) in the presence of DMA [5,24], which include two crystallographically-independent tetramers (AABB and CCDD); we have therefore trapped in the crystal two slightly different configurations of the tetramer corresponding to two distinct subpopulations, possibly providing two snapshots of the permeation process. The electron density is characterized by one strong peak at site 3 (**S3**^**c**^) in both AABB and CCDD tetramers, which has been modelled as a DMA ion (PDB ID: 4R7C) (Fig 1) [5]. Moreover, additional weaker electron density peaks in the cavity are observed just below (**S4**^**c**^**)** in the AABB tetramer and in the inner layer right above the site 1 (**S0**^**c**^) in the CCDD tetramer. These peaks could be interpreted as either water molecules or a partially occupied DMA cation (Fig 1).

**Fig 2:**
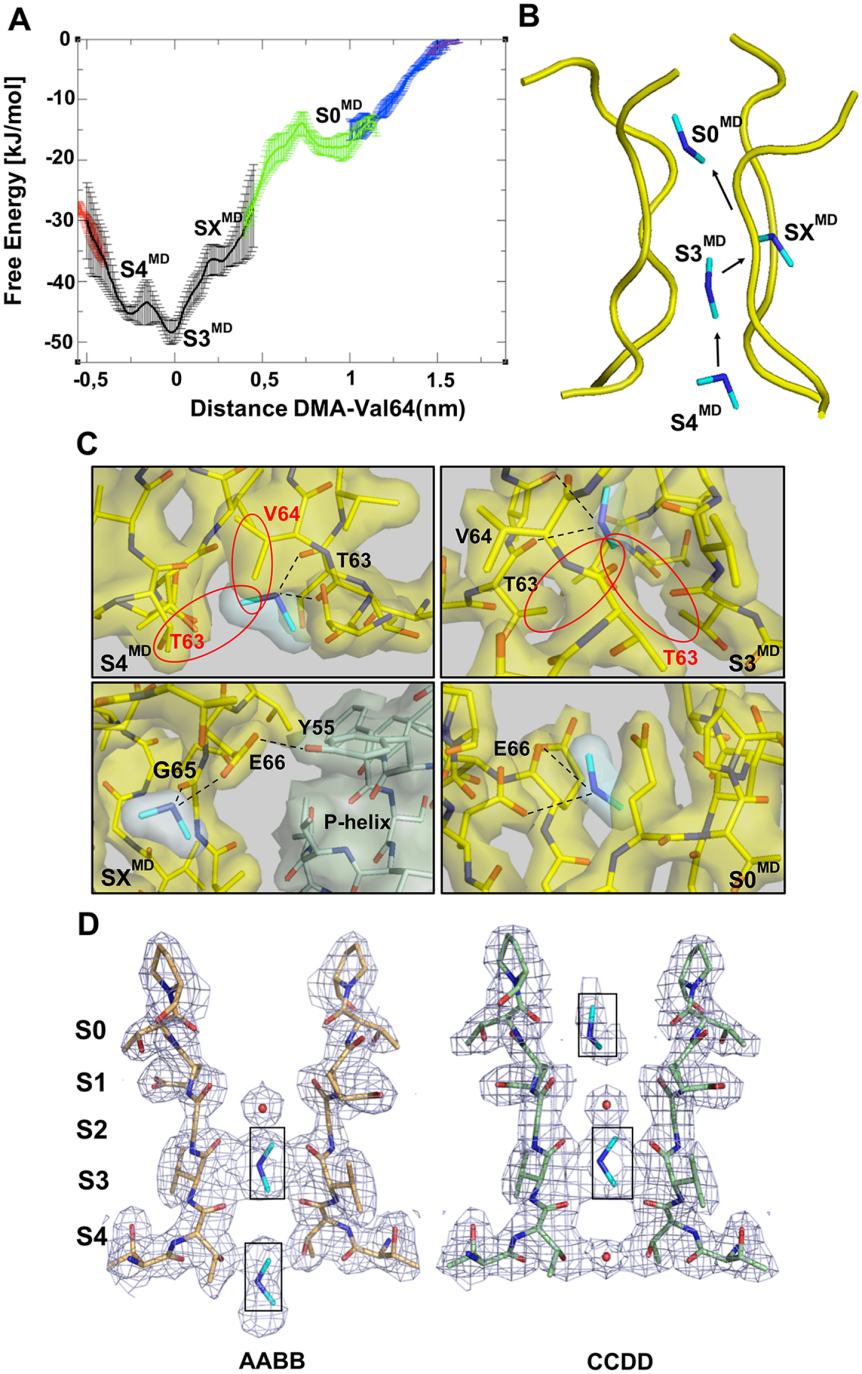
DMA permeation pathway. **(A)** Free Energy profile (kJ/mol) along the distance of the DMA from its binding site, represented by center of mass of Val64 (nm). The different colors indicate the runs in which the whole BE-META has been divided (See Materials and methods). The different free energy minima are indicated as S4^MD^, S3^MD^, SX^MD^ and S0^MD^. All the free energy profiles are shown with the relative error bars estimated by block analysis [29] **(B)** Position and interaction of the DMA ions in the different minima along the permeation pathway in the CNG-mimic selectivity filter, as modelled by MD. DMA is depicted in stick, while only the Cα trace of the selectivity filter is shown. **(C)** Close-up of the MD structures corresponding to the minima shown in (A-B). The residues forming hydrogen bonds with DMA (Thr63; Val64; Gly65; Glu66 and Tyr55) and the DMA itself have been drawn as sticks. The black dashed lines refer to H-bonds, while the red circles indicate the hydrophobic interactions. **(D)** Electron density map for the selectivity filter for of the AABB and CCDD tetramers (as in Fig 1) where the electron density peaks previously assigned to water molecules have been replaced by weakly bound DMA cations, based on the MD results.

To investigate the exact nature of the binding sites for DMA we performed Molecular Dynamics simulations of the CNG mimic embedded in a lipid membrane and solvated by water molecules [5]. As crystallography shows a clear occupancy inside the pore only for one DMA, we initially considered a single DMA. We first verified that, due to its hydrophobic nature, DMA in **S3**^**c**^ appears to be less hydrated than monovalent cations [15]. Indeed, the number of waters around the DMA in a sphere of 3Å is 1–2 during the entire long-term MD simulation (S1 Fig). DMA is stabilized in the **S3**^**c**^ site by both hydrogen bonding with Val64 carboxyl oxygen and hydrophobic interactions with the CH_3_ moiety of Thr63. To study in details DMA permeation through the pore, we then used the Bias Exchange-Metadynamics (BE-META) scheme [27] that allows computing the multidimensional free energy landscape of the system as a function of a set of Collective Variables (CVs) (see Materials and methods). The projection of the free energy along the vertical distance of the DMA from the Val64 residue corresponding to site **S3**^**c**^ in Fig 1—in the selectivity filter provided a description of the DMA progression along the channel (Fig 2 and S2 Fig).

Interestingly, the first free energy minimum (**S4**^**MD**^ in Fig 2A and 2B) corresponds to the weak electron density peak above site 4 observed in the crystal structure of the AABB tetramer. Molecular Dynamics indicates that a DMA ion in this position is stabilized by a bifurcated hydrogen bond with the hydroxyl group of two Thr63 and by hydrophobic interactions with the side chain of Val64 and the methyl groups of Thr63 (Fig 2C). As a consequence, the **S4**^**MD**^ site is asymmetrically disposed along the pore direction. The next site along the pore axis, **S3**^**MD**^ is the deepest free energy minimum and corresponds to the main DMA site identified in the crystal structure (**S3**^**c**^ in Fig 1) (Fig 2A and 2B). Due to the two-fold symmetry axis of the crystallographic tetramer, in the crystal structure the DMA ion was modeled in a “horizontal” configuration, whereas MD simulation, having no symmetry constrains, shows a more “vertical” configuration, aligned with the selectivity filter. In this position, the DMA is engaged in a bifurcated hydrogen bond with both the carboxyl oxygen of Val64 and the Thr63 hydroxyl group, while one of its methyl groups still points towards the CH_3_ moieties of two of the Thr63 residues in the filter (Fig 2C). A site “off-axis”, which does not coincide with any of the canonical binding sites along the pore, is also visualized (**SX**^**MD**^ in Fig 2A and 2B), where the DMA forms a hydrogen bond with the Gly65 of a single subunit; in this free energy minimum the DMA is stabilized by a bifurcated hydrogen bond with the Gly65 and Glu66 carboxylates. As previously observed, Glu66 has an important role in CNG channels, being engaged in an intrasubunit interaction with Tyr55 residue in the P-helix [5]. Glu66 side chain significantly shifts from the crystallographic position, moving away from the pore axis and bringing the DMA in the last free energy minimum (**S0**^**MD**^ in Fig 2A–2C). This last site corresponds to the weaker electron density peak in the inner layer right above the site **S1** (**S0**^**c**^) in the CCDD tetramer of the crystallographic molecule (Fig 1).

The MD results resolve the ambiguities of the crystallographic data concerning the interpretation of the electron density peaks in the selectivity filter shown in Fig 1. These peaks correlate very well with **S4**^**MD**^, **S3**^**MD**^ and **S0**^**MD**^ (Fig 2D). The global Free Energy minimum (**S3**^**MD**^) corresponds to a density peak that can be unambiguously assigned to DMA. The site **S4**^**MD**^ is disfavored by 3 kJ/mol with respect to **S3**^**MD**^ and is therefore occupied only occasionally by DMA. Previous studies have suggested that the ring of Thr360 (equivalent to Thr63 in the CNG mimic) forms a binding site for intracellular cations [28] and that Na^+^ inward current is reduced by the presence of intracellular ammonium derivatives [3]. To assess the contribution of Thr360 in DMA permeation we studied the DMA current flowing through Thr360A mutants at different voltages. These records clearly show that this mutation strongly affects the DMA current-voltage relationship (S3 Fig), suggesting an important role of these Thr in DMA coordination. Finally, site **SX**^**MD**^ corresponds to a small free energy minimum, significantly less stable than the other minima. To sum up, BE-META, combined with crystallography, clearly identifies **S3**^**MD**^—equivalent to **S3**^**c**^ —as the main binding site for DMA. However, it is also points to **S4**^**MD**^—equivalent to **S4**^**c**^—as an important additional binding site with a lower, but significant, occupancy than S3^MD^. The presence of this second free energy minimum prompted us to investigate the behavior of the channel in the presence of a second DMA.

### DMA in S3^MD^ is destabilized by a second DMA

**Fig 3:**
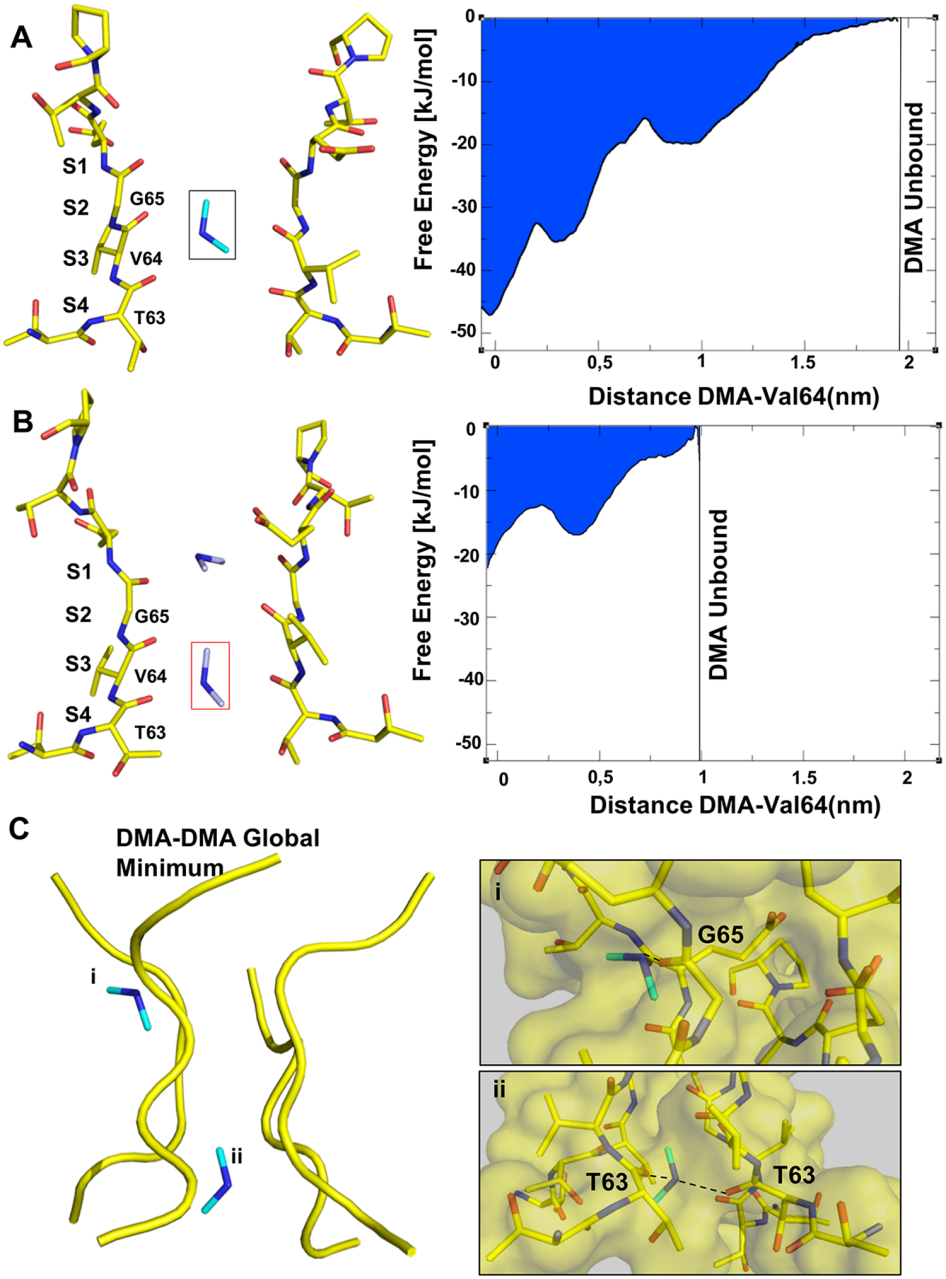
The presence of a second DMA destabilizes the first one. **(A)** and **(B) Left side**: Representative structures of the MD simulations performed in the presence of a single DMA in S3 **(A)** and two DMA (**B)**. For simplicity only the filter domain (Thr62-Pro68) of the NaK2CNG channel is shown. DMA has been drawn as cyan sticks while the amino acids residues as yellow sticks. (A) and (B) **Right side**: Free Energy profile (kJ/mol) along the distance of the DMA from its binding site, represented by center of mass of Val64 (nm), in the case of a single DMA (A) and in the DMA-DMA system (B). **(C)** Position and interaction of the DMA ions in the global minimum along the permeation pathway in the CNG-mimic selectivity filter, as modelled by MD. DMA is depicted in stick, while only the Cα trace of the selectivity filter is shown. In **i)** and **ii)** panels, close-up of the MD structures corresponding to the DMA-DMA global minimum. The residues and the DMA itself have been drawn as sticks. The residues forming hydrogen bonds with DMA are indicated (Thr63 and Gly65). The black dashed lines refer to H-bonds.

To verify whether two DMA could simultaneously fit inside the CNG pore with a full occupancy, we performed several unbiased MD simulations with one DMA in S3—corresponding to S3^MD^—and the other in S2 (Fig 3). In this new configuration, the DMA in S3 is pushed towards the intracellular side, due to their electrostatic repulsion. The DMA reaches a different position to the one observed in the crystallographic structures and in the global free energy minimum (Fig 3 and S4 Fig). Indeed, the number of contacts between the N of the DMA and the hydroxyl oxygens of the Thr63 is significantly higher in the case of the DMA-DMA configuration then for the single DMA (S3 Fig). To gain insight into the effect that a second DMA has on the DMA in S3, we estimated the free energy landscape experienced by the system during the DMA-DMA configuration using the BE-META scheme [27] (S5 Fig) (see Materials and methods). Remarkably, the projection of the free energy along the vertical distance of the DMA from the Val64 residue—corresponding to both **S3**^**MD**^ and **S3**^**c**^—in the selectivity filter revealed that the presence of a second DMA completely changes the free energy landscape. Indeed, the presence of the second DMA decreases the depth of the well by 25 kJ/mol (Fig 3B-right panel) compared to the single DMA system (Fig 3A). When a single DMA is in the channel, it needs to move to a distance of 2 nm from Val64 to become unbound from the selectivity filter and to become free to diffuse in the cytoplasm; in the presence of two ions, the DMA becomes unbound at a distance of 1 nm from Val64 (Right panels in Fig 3A and 3B). Moreover, when two DMA are present inside the channel, there is a complete rearrangement of the position of the binding sites with one global minimum corresponding to a DMA in a site “off-axis” where, similarly to the **SX**^**MD**^ site described in the Fig 2C, the DMA forms a hydrogen bond with the Gly65 of a single subunit (i panel in Fig 3C); and the second DMA stabilized by a bifurcated hydrogen bond with the hydroxyl group of two Thr63 (ii panel in Fig 3C). Taken together these data highlight that the presence of a second DMA destabilizes the first one and strongly affects the structure of the channel, completely changing the free energy landscape.

### Electrophysiological analysis of DMA permeation through CNGA1 channels

**Fig 4:**
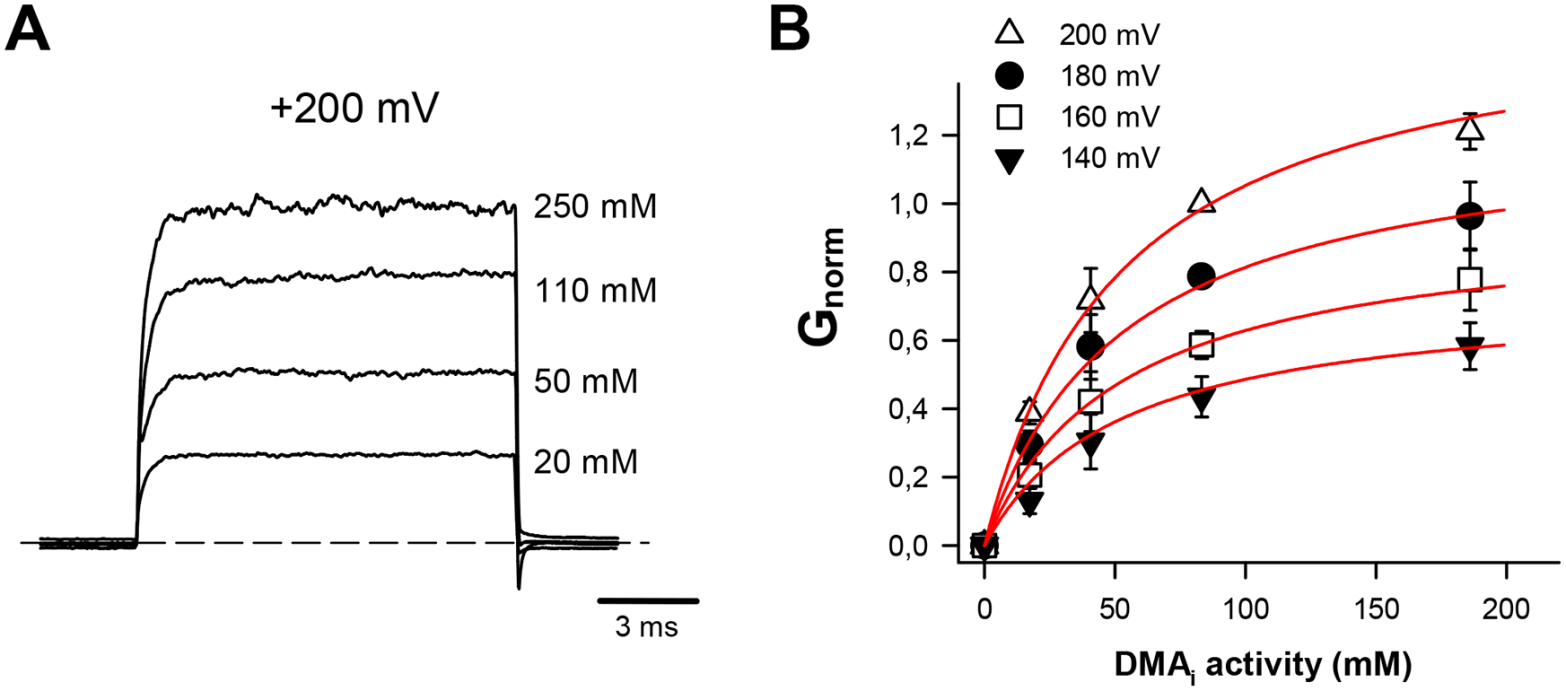
Currents activated by 1 mM cGMP in the presence of different voltage commands and amounts of DMA. **(A)** Representative currents observed in the presence of different amounts (indicated in the figure) of intracellular DMA (DMA_i_) at +200 mV (holding potential of 0 mV). The dashed line indicates the zero-current level. Currents are normalized to the mean current observed in the presence of 110 mM DMA_i_ and the solution filling the patch pipette contained 110 mM DMA. **(B)** Dependence of the cGMP-gated conductance on the ionic activity of DMA_i_ at +140 (filled triangles), +160 (open squares), +180 (filled circles) and +200 mV (open triangle). Conductances are normalized to the conductance observed at +200 mV in the presence of 110 mM DMA_i_. Each point is the average obtained from at least three patches. These data have been fitted with the Eq (3) obtaining an apparent Kd = 52 mM. Data are presented as mean +/- SD (n = 4).

In order to further validate the picture emerging from MD simulations we then performed electrophysiological measurements aimed at estimating the affinity of the CNGA1 channel for DMA. We prepared a patch with 110 mM DMA inside the patch pipette and we varied the concentration of DMA in the bathing medium—corresponding to the intracellular side of the membrane—from 0 to 250 mM (Fig 4). Fig 4A shows representative currents observed at 200 mV when the concentration of DMA in the bath was 20, 50, 110 and 250 mM, respectively. The cGMP activated current was measured as the difference between the current recorded in the presence of cGMP and its absence. Fig 4B shows the values of the observed normalized conductance at different voltages between +140 and +200 mV as a function of the DMA activity. At these high membrane potentials, the outward current is carried by DMA ions moving from the bath toward the patch pipette, and backward crossing is assumed to be negligible. We simultaneously fitted these data with the Michaelis-Menten equation [7], which is derived assuming that the channel is occupied by at most one ion at a time. As illustrated in Fig 4B, the match between the experimental data and this model is almost perfect. However, the binding affinity estimated by this fit is of 52 mM. Clearly this number is not consistent with the free energy profile reported in Fig 2, which, in the case of a single DMA ion, is characterized by the presence of a free energy minimum whose depth would imply a binding affinity in the low nanomolar range.

### A model for DMA permeation through CNG channels

In order to understand the reason for this discrepancy, we considered a generalization of the Michaelis Menten theory, in which a channel can be simultaneously occupied by two ions. We will show that this model, *in the presence of a significant interaction between the ions*, predicts that the relation between the current J and the concentration of the permeating ion X_in_ has the same functional form of the Michaelis Menten equation, but with a different half-activation constant K_1/2_.

In the standard Michaelis Menten the channel is characterized by two states: empty (p_E_) and occupied (p_O_), with *p*_*E*_ + *p*_*O*_ = 1.

**Fig 5:**
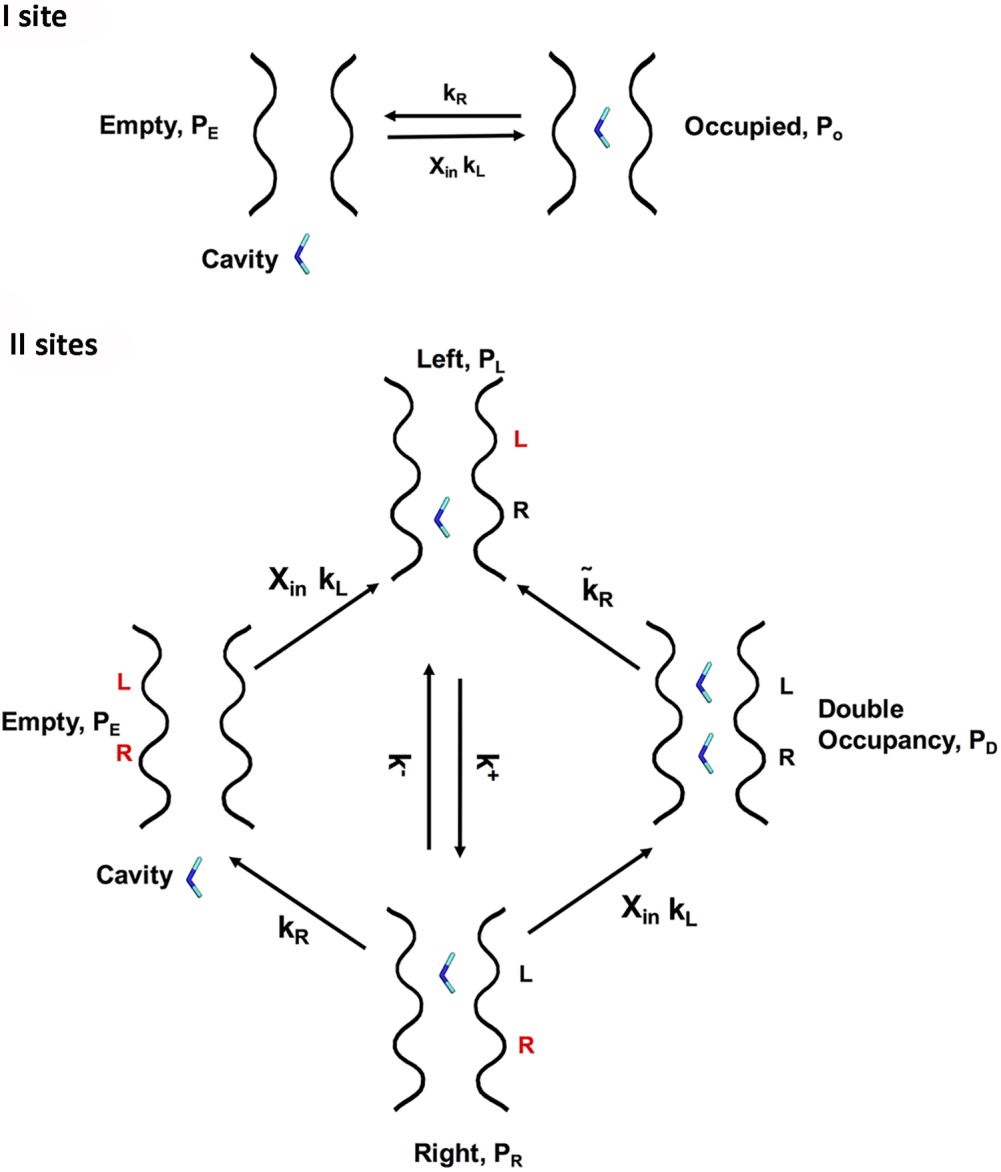
Model for the DMA permeation through the CNGA1 channel. The first scheme (I site) describes a simple model of DMA permeation in which there is a single binding site, while the second scheme (II sites) depicts a different scenario with the CNGA1 channel occupied simultaneously by two ions referred to as right (R) and left (L). P_E_ is the probability that the channel is empty, P_O_ refers to the channel occupied; P_L_ is the probability that the channel is occupied in the left site, P_R_ is occupied in the right side, P_D_ when the channel is occupied by two ions. k_L_ is the rate for the transition in which the ion enters into the channel, while k_R_ for the transition in which the ion leaves the channel crossing the barrier towards the right. The rate $\tilde{k_{R}}$ is associated to a transition between the state with the Double Occupancy and the state with a single ion in the Left side. k^+^ is the transition rate from the binding site at the left to that at the right and k^-^ is the corresponding reverse rate. The transition rates k_R_, k_L_, k^+^ and k^-^ are estimated from the Free Energy profile computed with a single DMA inside the pore (see right panel in Fig 3A) and the transition rate $\tilde{k_{R}}$ is estimated from the Free Energy profile computed with a second DMA inside the pore (see right panel in Fig 3B).

Under the assumption that backward crossing can be neglected, i.e. that ions can move only from the intracellular to the extracellular medium, the model is therefore fully defined by two rates: the rate k_L_ for the transition in which the ion enters into the channel, and the rate k_R_ for the transition in which the ion leaves the channel crossing the barrier towards the right (Fig 5).

At the stationary state, we have:

$$J=k_{L\;}{[X}_{in}]p_{e}$$

$$0=k_{L}{[X}_{in}]p_{e}-k_{R}p_{O}$$ (1)

where [X_in_] is the concentration of the ion X_in_ in the intracellular medium (Fig 5). From these equations, we obtain the usual Michaelis-Menten equation [7]:

J=kL[Xin][Xin]kLkR+1 (2)

We now consider the case in which the channel can be occupied by two ions, in two binding sites at the left (L) and the right (R). The channel can now exist in four states: (i) empty (with probability p_E_); (ii) occupied by one ion in the right site (probability p_R_); (iii) occupied by one ion in the left site (probability p_L_) and (iv) occupied by two ions (probability p_D_). The allowed transitions between these states are as in Fig 5. After some algebra reported in S1 Appendix we obtain that the flux *J* of ions of concentration X_*in*_ from the intracellular to the extracellular medium is given by

$$J=\frac{{[X}_{in}]k_{L}k^{+}\;\tilde{k_{R}}({[X}_{in}{]k}_{L}+k_{R})}{(k^{+}+\tilde{k_{R}})[{X_{in}]2k}_{L}^{2}+\tilde{k_{R}}\;(k_{R}+k^{-}+k^{+})[X_{in}]k_{L}+k_{R}k^{+}\tilde{k_{R}}}$$ (3)

here k^+^ is the transition rate from the binding site at the left to that at the right and k^-^ is the corresponding reverse rate. The rate $\tilde{k_{R}}$ is associated to a transition between the state with double occupancies (D) and the state with a single ion occupying the left side (L). The rates k^+^, k^-^ and k_R_ can be estimated from the free energy profile reported in Fig 2. Indeed, assuming that the rate satisfies Arrenius law, and assuming that the kinetic prefactor is the same for all the rates, we have k+ ≈exp (-0.5), k- ≈exp (-1.5) and k_R_ ≈ exp (-15) (all in the units of the kinetic prefactor). We have seen that the rate $\tilde{k_{R}}$ is approximately equal to exp (-7.5) significantly smaller than k^+^ and k^-^, but much higher than k_R_, due to the repulsion between the two ions. If we neglect the terms proportional to k_R_, Eq 3 becomes

$$J=\frac{k_{L}k^{+}}{\left(k^{-}+k^{+}\right)}\;\frac{{[X}_{in}]}{\frac{(k^{+}+\tilde{k_{R}})k_{L}}{(k^{-}+k^{+})\tilde{k_{R}}}{[X}_{in}]+1}$$ (4)

Remarkably, the functional form of the dependence of the current on the concentration is exactly the same for this model and for the model with a single site (see Eq 2). Therefore, the two scenarios cannot be distinguished based on the dependence of the current of the concentration. From Eq (4) the term

$$\frac{(k^{+}+\tilde{k_{R}})k_{L}}{(k^{-}+k^{+})\tilde{k_{R}}}$$ (5)

plays the role of the inverse of the of the concentration of ions causing half of the maximal current. Inserting in this equation the estimates of the rates obtained from the metadynamic profiles (see legend of Fig 5), we find that this concentration has the value of 0.6 mM. This value is close to what seen experimentally in Fig 4, where a global fitting of the data obtained at different voltages yielded an apparent Kd of 52 mM.

Summarizing, when two DMA ions are simultaneously present inside the pore, the interaction between the ions lowers the exit rate by several orders of magnitude bringing in qualitative agreement the experimentally measured half activation value with that obtained from simulation. The residual discrepancy can be ascribed to the several approximations that we have done to estimate the rates, and to the inaccuracy of the force field used for simulating the channel presumably due to the assumption that the charges of the atoms are fixed and do not depend on the environment.

In summary, by combining electrophysiology and Molecular Dynamics (MD) simulations, we uncover an unusual molecular mechanism underlying the permeation of large organic cations through the CNGA1 channels. We find that the free energy landscape associated to the translocation of a single dimethylammonium (DMA) through the CNGA1 pore is characterized by the presence of a few small local minima in a single very deep free energy well. This contrasts sharply with the free energy landscape for monovalent alkali cations, which are normally characterized by the presence of at least one free energy barrier [19]. We identified the molecular mechanisms leading to the DMA permeation through the CNGA1 channel: the presence of a second DMA changes completely the free energy landscape, leading to a destabilization of the DMA-channel complex. In agreement with experimental measurements, the free energy well depth is reduced by about half when two DMA occupy simultaneously the selectivity filter. Like in other multi-ion channels, including the Ca^2+^ channels [16,17] and CorA channels at high Mg^2+^ concentration [30], the permeation of DMA is due to the interaction between the ions. However, in the CNGA1 this interaction induces a significant change in the free energy landscape: the binding sites observed in the presence of a single DMA ion are not observed when two ions are present. This is ultimately a consequence of the significant flexibility of the CNGA1. This effect is accurately described by molecular simulations. Indeed, a model in which the ions move in a single file on a pre-sculptured free energy landscape with a sequence of free energy minima is clearly not adequate for describing the permeation process of DMA through the CNGA1. This finding has a remarkable physiological relevance since it discloses an unexpected mechanism for the permeation of large cations through the CNGA1 channel and, possibly, through other voltage-gated channels.

## Materials and methods

### Protein expression and purification

Protein expression and purification for the NaK2CNG-E plasmid was performed as previously described [5]. Briefly, the plasmid coding for the NaK2CNG-E chimera fused to a C-terminal hexahistidine tag was transformed into *Escherichia coli* XL1B, and following expression at 25°C the proteins were extracted in 50 mM Tris•HCl pH 8, 100 mM DMA•HCl, 40 mM n-decyl-β-D-maltoside (DM) (Anagrade) and purified by affinity chromatography on a Talon resin, followed by size esclusion chromatography on a Superdex-200 gel filtration column (GE Healthcare) in 20 mM Tris • HCl pH 8.0, 5 mM DM and 100 DMA• HCl.

### Protein Crystallization and Structure Determination

Purified NaK2CNG-E was concentrated to 20 mg/mL and crystallized using the sitting drop vapor diffusion method at 20°C by mixing equal volumes of protein and reservoir solution containing 40–44% (±)-2-methyl-2,4-pentanediol (MPD), 100 mM of MES pH 6.5 and 25mM Glycine. Crystals belong to space group P222_1_ with cell dimension a = 67.62, b = 67.68Å and c = 89.90Å. All crystals were flash-frozen in liquid nitrogen and X-ray diffraction data were collected at 100K at Eettra XRD1 beamline at 1 Å. Data reduction was performed as previously described [5]. Briefly, iMOSFLM [31], XDS [32,33] and the CCP4i suite [34] were used. Resolution cut-off was chosen following I/σI criterion [35]. The dataset exhibited merohedral twinning [36]. The structure was determined by molecular replacement using the published K^+^ complex structure (PDB: 3K0D) with selectivity filter region omitted as an initial search model. Repeated cycles of refinement using REFMAC5 [37] and model building using Coot [38] was carried out. Figs 1, 2 and 3 were prepared using PyMOL [39]. One-dimensional electron density profiles were obtained by sampling the electron density maps along the central axis.

### Models and simulations

The starting point of this study was the structure of the NaK2CNG-E obtained from our previous study [5]. The model of the chimera NaK2CNG-E was built using the chain B (residues from 19 to 113) of the 2 Å resolution crystal structure soaked with Na+ ions (PDB accession code 3K0G) [24]. The protein was embedded in a pure, pre-equilibrated 1-palmitoyl-2-oleilphosphatidylcholine (POPC) lipid model (kindly supplied by T. A. Martinek) [40] using the g_membed4 tool of Gromacs and then it was oriented following OPM5 database model. Afterward the system was neutralized and solvated with TIP3P model6 water molecules (76305 total atoms in a box size of 92.8 91.9 87.5 Å3). The system was prepared with a single DMA in the strongest binding site identified by the crystal structure. The simulations were performed in periodic boundary conditions at 300 K using the Nose-Hoover thermostat7 and Parrinello-Rahman barostat with a semisotropic pressure coupling type and a time step of 2 fs. Position restraints of atoms were fixed with a force constant (K) equal to 1000 kJ mol^-1^ nm^-2^. We used GROMACS410 package with Amber0311 force field for protein and GAFF12 for the membrane. The equilibration was performed in three stages: (1) the system was heated for 2.5 ns with protein backbone and DMA fixed, while sidechains were left free to move; (2) 5.2 ns were run using position restraints only for the selectivity filter and the DMA. In the first stage we used the NPT ensemble, while in the second one a surface tension equal to 600.0 bar*nm2 was added. (3) For the next 1 ns the membrane area was kept constant. A configuration taken from this step was used as a starting point for a molecular dynamics (MD) simulation of 98 ns. The same procedure was followed with the 2 DMA system.

### Bias-Exchange Metadynamics

In order to better explore the free energy surface associated to the DMA permeation pathway, we performed a Bias-Exchange Metadynamics (BE-META) simulation of 450 ns (50 ns x 9 walkers), using the Plumed package [41]. The Collective Variables used are: 1) the distance of the DMA from its binding site, represented by center of mass of Val64; 2) the distance between Cα of E66 and C (of carboxylic group) of Glu66 in the opposite monomer; 3) the coordination number of the ions with the two oxygens of the carboxylic group of E66s; 4) the distance between Cα of Gly65 in the opposite monomer; 5) the radius of gyration of the Gly65 residues; 6) the distance between Cα of Pro68 in the opposite monomer; 7) the radius of gyration of the Pro68 residues. In the case of the 2 DMA system, we considered also the distance of the second DMA (DMA in S1 site in Fig 4A) from the center of mass of Val64 as Collective Variable. We have divided the whole BE-META run in 4 separate runs, each corresponding to the different colors shown in Fig 2, in order to enhance the convergence of the system following a standard procedure of weighted-histogram procedure [42]. The 4 Plumed input files containing the exact definition of these collective variables have been provided as S2 Appendix

All structural and free energy analyses were performed using METAGUI, a VMD interface for analyzing metadynamics and MD simulations [43]. The structures generated during such a simulation are clustered together into a set of microstates (i.e. structures with similar values of the collective variables) and their relative free energies are then computed by a weighted-histogram procedure, METAGUI returns configurations which are representative of ensemble averages of the corresponding microstates [43].

### Ethical approval

All studies were approved by the SISSA’s Ethics Committee according to the Italian and European guidelines for animal care (d.l.26, March 4th 2014 related to 2010/63/UE and d.l. 116/92; 86/609/C.E.). Oocytes were harvested from female *Xenopus laevis* frogs (‘Xenopus express’ Ancienne Ecole de Vernassal, Le Bourg 43270, Vernassal, Haute-Loire, France) using an aseptic technique or, if necessary, purchased from Ecocyte Bioscience (Am Förderturm, 44575, Castrop-Rauxel, Germany). *Xenopus laevis* were kept in tanks—usually 6–8 animals per tank—and were exposed to a 12/12 hours dark/light cycle. All *Xenopus laevis* surgeries were performed under general anesthesia, obtained by immersion in a 0.2% solution of tricaine methane sulfonate (MS-222) adjusted to pH 7.4 for 15–20 min. Depth of anesthesia was assessed by loss of the righting reflex and loss of withdrawal reflex to a toe pinch. After surgery, animals were singly housed for 48 h. Frogs were monitored daily for 1 week post-operatively to ensure the absence of any surgery-related stress. Post-operative analgesics were not routinely used. Considering the simplicity of the procedure, the lack of complications, the effectiveness of anesthetic regimen and the reduction in the number of animals likely to be used compared to the number that would be required if only one surgery were permitted, multiple surgeries on a single animal were performed. Individual donors were used up to five times, conditional upon the health of an individual animal. Recovery time between oocyte collections from the same animal was maximized by rotation of the frogs being used. A minimum recovery period of 1 month was ensured between ovarian lobe resection from the same animal to avoid distress. Evidence of surgery-related stress resulted in an extended rest period based on recommendations from the veterinary staff. After the fifth terminal surgery frogs were humanely killed through anesthesia overdose via 2 h of immersion in a 5 g/l MS-222 solution adjusted to pH 7.4.

### Oocyte preparation and chemicals

Bovine CNGA1 cRNA were injected into *Xenopus laevis* oocytes. Oocytes were prepared as described [44]. Injected eggs were maintained at 18°C in a Barth solution supplemented with 50 μg/ml gentamycin sulfate and containing (in mM): 88 NaCl, 1 KCl, 0.82 MgSO_4_, 0.33 Ca(NO_3_)_2_, 0.41 CaCl_2_, 2.4 NaHCO_3_ and 5 Tris-HCl, pH 7.4 (buffered with NaOH). During the experiments, oocytes were kept in a Ringer solution containing (in mM): 110 NaCl, 2.5 KCl, 1 CaCl_2_, 1.6MgCl_2_ and 10 Hepes, pH 7.4 (buffered with NaOH). Usual salts and reagents were purchased from Sigma Chemicals (St Louis, MO, USA).

### Recording apparatus

cGMP-gated currents from excised patches were recorded with a patch-clamp amplifier (Axopatch 200; Axon Instruments Inc., Foster City, CA, USA), 2–6 days after RNA injection, at room temperature (20–24°C) [45]. The perfusion system allowed a complete solution change in less than 0.1 seconds [44,45]. Macroscopic current recordings were obtained with borosilicate glass pipettes which had resistances of 2–5 MOhm in symmetrical standard solution. The standard solution on both sides of the membrane consisted of (in mM) 110 DMACl, 10 Hepes and 0.2 EDTA buffered with tetramethylammonium hydroxide (pH 7.4). The ion composition of the bath solutions was similar except 110 mM-DMACl was substituted as specified in the figure legends. We used Clampex version 10.0 for data acquisition. Recordings were low-pass filtered at 5 kHz and sampled at 20 kHz kHz. The activity coefficients γ_i_ were calculated according to Debye-Huckel equation (for DMACl concentrations below 100 mM)

$$log\gamma_{i}=\frac{-0.509z_{i}^{2}\sqrt{I_{c}}}{1+(3.28d_{i}\sqrt{I_{c})}}$$

or Davies equation (for higher DMACl concentrations)

$$log\gamma_{i}=-0.509(\frac{\sqrt{I_{c}}}{1+\sqrt{I_{c}}}-0.3I_{c})$$

where I_c_ is the ionic strength of the solution, z_i_ is the charge (+1) and d_i_ is the ion size parameter (0,35 nm) for DMA [46]. No statistical methods were used to predetermine sample sizes that are similar to those reported in previous publications [47–49]. We normally excluded data when we lost the patch during the experiments, when the level of expression was too low and we could not distinguish the noise from random channel openings from electrical noise due to an unstable patch and /or from spurious electrical noise. We kept only data obtained during experiments in which the amplitude of the seal (i.e. the current evoked by voltage pulses in the absence of cGMP) was stable. Representative electrical recordings are shown as well as descriptive statistics where data are presented as mean +/- SD. n indicates the number of excised patches.

## Supporting information

S1 Fig Hydration of the DMA in the CNG pore. Superimposition of 10 instantaneous configurations taken from MD simulations showing the DMA coordination in **S3**^**c**^. The black dashed line refers to H-bonds, while the red dashed circle indicates the hydrophobic interactions. The average number of water coordinating DMA is about 1.5. For simplicity only the residues 62–66 are shown for two opposite subunits in the tetramer. DMA cation is shown as cyan sticks and well-ordered waters as red spheres.
(TIF)

S2 Fig Evolution of the free energy profiles along time. Free energy profiles (kj/mol) as a function of the distance of the DMA from its binding site, represented by center of mass of Val64 (nm). Different colors refer to different times.
(TIF)

S3 Fig Currents activated by 1 mM cGMP for recombinant WT CNGA1 channel and pore mutants T359A and T360A in the presence of symmetrical DMA. **(A)** Sequence alignment in the selectivity filter of the NaK2CNG-E, bCNGA1, hCNGA1, hCNGA2 and hCNGA3 channels. In red, the Thr which have been mutated in Ala. **(B)** Structure of the NaK2CNG-E selectivity filter (PDB ID: 4R7C) showing the position of the T62 and T63 residues. (**C-E)** Representative currents observed in the presence of symmetrical solutions (110 mM) of DMA for WT channel **(C)**, T359A **(D)** and T360A **(E)** mutants. Voltage commands are from -200 to +200 mV in 20 mV steps (holding potential of 0 mV). **(*F*)** Normalized I–V relationship for the recordings in *A-C* (black dots, open squares, and open triangles refer to WT channel, T359A, and T360A mutants, respectively.
(TIF)

S4 Fig DMA is shifted towards T63 in the presence of a second DMA. Right side: The tetrameric structure of a single DMA and 2 DMA complexes are superimposed and only the residues 62–68 are shown for the opposite subunits in the tetramer. The DMA complex is colored in yellow, while DMA-DMA complex in wheat. Changes in the position of the DMA can be observed. **Left side**: The contact numbers between the N of the DMA and the O of the hydroxyl group of T63 in both single DMA and DMA-DMA complex structures.
(TIF)

S5 Fig DMA permeation pathway in the DMA-DMA configuration. Free Energy profile (kJ/mol) of the DMA-DMA configuration along the distance of the DMA from its binding site, represented by center of mass of Val64 (nm). The free energy profile is shown with the relative error bars estimated by block analysis [29].
(TIF)

S1 Appendix A model for DMA permeation through the CNGA1 channels. A detailed explanation of the model reported in Fig 5.
(PDF)

S2 Appendix Plumed files used in the BE-META scheme. Here are reported the Plumed files used to obtain the Free Energy profile reported in Fig 2.
(PDF)

## References

[1] Kaupp UB, Seifert R. Cyclic nucleotide-gated ion channels. Physiol Rev. 2002;82: 769–824. 10.1152/physrev.00008.2002 12087135

[2] Zheng J, Trudeau MC. Handbook of Ion Channels [Internet]. Journal of Chemical Information and Modeling. 2015 10.1201/b18027

[3] Picco C, Menini A. The Permeability of the Cgmp-Activated Channel to Organic Cations in Retinal Rods of the Tiger Salamander. J Physiol. 1993;460: 741–758. 10.1113/jphysiol.1993.sp019497 7683718 PMC1175239

[4] Arcangeletti M, Marchesi A, Mazzolini M, Torre V. Multiple mechanisms underlying rectification in retinal cyclic nucleotide-gated (CNGA1) channels. Physiol Rep. 2013;1: 1–19. 10.1002/phy2.148 24400150 PMC3871463

[5] Napolitano LMR, Bisha I, De March M, Marchesi A, Arcangeletti M, Demitri N, et al A structural, functional, and computational analysis suggests pore flexibility as the base for the poor selectivity of CNG channels. Proc Natl Acad Sci U S A. 2015;112: E3619–28. Available: http://www.pnas.org/content/112/27/E3619.short 26100907 10.1073/pnas.1503334112 PMC4500290

[6] Hille B, Schwarz W. Potassium channels as multi-ion single-file pores. J Gen Physiol. 1978;72: 409–442. 10.1085/jgp.72.4.409 722275 PMC2228548

[7] Hille B. Ion Channels of Excitable Membranes. 3rd edition Sinauer Associates, Sunderland 2001 10.1007/3-540-29623-9_5640

[8] Laio A, Torre V. Physical origin of selectivity in ionic channels of biological membranes. Biophys J. 1999;76: 129–148. 10.1016/S0006-3495(99)77184-5 9876129 PMC1302506

[9] Åqvist J, Luzhkov V. Ion permeation mechanism of the potassium channel. Nature. 2000;404: 881–884. 10.1038/35009114 10786795

[10] Bernèche S, Roux B. Energetics of ion conduction through the K+ channel. Nature. 2001;414: 73–77. 10.1038/35102067 11689945

[11] Noskov SY, Bernéche S, Roux B. Control of ion selectivity in potassium channels by electrostatic and dynamic properties of carbonyl ligands. Nature. 2004;431: 830–834. 10.1038/nature02943 15483608

[12] Furini S, Domene C. Atypical mechanism of conduction in potassium channels. Proc Natl Acad Sci. 2009;106: 16074–16077. 10.1073/pnas.0903226106 19805261 PMC2752519

[13] Bezanilla F, Armstrong CM. Negative conductance caused by entry of sodium and cesium ions into the potassium channels of squid axons. J Gen Physiol. 1972;60: 588–608. 10.1085/jgp.60.5.588 4644327 PMC2226091

[14] Doyle DA, Cabral JM, Pfuetzner RA, Kuo A, Gulbis JM, Cohen SL, Chait BT, Mackinnon R. The Structure of the Potassium Channel: Molecular Basis of K+ Conduction and Selectivity. Science. 1998;280: 69–77. 10.1126/science.280.5360.69 9525859

[15] Noskov SY, Roux B. Importance of hydration and dynamics on the selectivity of the KcsA and NaK channels. J Gen Physiol. 2007;129: 135–143. 10.1085/jgp.200609633 17227917 PMC2154357

[16] Almers W, Mccleskey EW. Non-Selective Conductance in Calcium Channels of Frog Muscle: Calcium Selectivity in a Single-File Pore. J Physiol. 1984;353: 585–608. 10.1113/jphysiol.1984.sp015352 6090646 PMC1193323

[17] Hess P, Tsien RW. Mechanism of ion permeation through calcium channels. Nature. 1984;309: 453–456. 10.1038/309453a0 6328315

[18] Korn SJ, Ikeda SR. Permeation selectivity by competition in a delayed rectifier potassium channel. Science. 1995;269: 410–2. Available: http://www.ncbi.nlm.nih.gov/pubmed/7618108 7618108 10.1126/science.7618108

[19] Hodgkin AL, Keynes RD. The potassium permeability of a giant nerve fibre. J Physiol. 1955;128: 61–88. 10.1113/jphysiol.1955.sp005291 PMC1365755 14368575

[20] Sauer DB, Zeng W, Raghunathan S, Jiang Y. Protein interactions central to stabilizing the K. PNAS. 2011; 1–6. www.pnas.org/cgi/doi/10.1073/pnas.1111688108 21933962 10.1073/pnas.1111688108 PMC3189067

[21] Medovoy D, Perozo E, Roux B. Multi-ion free energy landscapes underscore the microscopic mechanism of ion selectivity in the KcsA channel. Biochim Biophys Acta—Biomembr. 2016;1858: 1722–1732. 10.1016/j.bbamem.2016.02.019 26896693 PMC4939264

[22] Liu S, Lockless SW. Equilibrium selectivity alone does not create K + -selective ion conduction in K + channels. Nat Commun. 2013;4 10.1038/ncomms3746 24217508

[23] Lockless SW. Determinants of cation transport selectivity: Equilibrium binding and transport kinetics. J Gen Physiol. 2015;146: 3–13. 10.1085/jgp.201511371 26078056 PMC4485025

[24] Derebe MG, Sauer DB, Zeng W, Alam A, Shi N, Jiang Y. Tuning the ion selectivity of tetrameric cation channels by changing the number of ion binding sites. Proc Natl Acad Sci. 2011;108: 598–602. 10.1073/pnas.1013636108 21187421 PMC3021048

[25] Derebe MG, Zeng W, Li Y, Alam A, Jiang Y. Structural studies of ion permeation and Ca2+ blockage of a bacterial channel mimicking the cyclic nucleotide-gated channel pore. Proc Natl Acad Sci U S A. 2010;108: 592–597. www.pnas.org/cgi/doi/10.1073/pnas.1013643108 21187429 10.1073/pnas.1013643108 PMC3021057

[26] Zhou Y, Morais-Cabral JH, Kaufman A, Mackinnon R. Chemistry of ion coordination and hydration revealed by a K+ channel-Fab complex at 2.0 Å resolution. Nature. 2001;414: 43–48. 10.1038/35102009 11689936

[27] Piana S, Laio A. A Bias-Exchange Approach to Protein Folding. J Phys Chem B. 2007;111: 4553–4559. 10.1021/jp067873l 17419610

[28] Marchesi A, Mazzolini M, Torre V. A ring of threonines in the inner vestibule of the pore of CNGA1 channels constitutes a binding site for permeating ions. J Physiol. 2012;590: 5075–5090. 10.1113/jphysiol.2012.238352 22869010 PMC3497564

[29] Marinelli F, Pietrucci F, Laio A, Piana S. A kinetic model of Trp-cage folding from multiple biased molecular dynamics simulations. PLoS Comput Biol. 2009;5 10.1371/journal.pcbi.1000452 19662155 PMC2711228

[30] Dalmas O, Sandtner W, Medovoy D, Frezza L, Bezanilla F, Perozo E. A repulsion mechanism explains magnesium permeation and selectivity in CorA. Proc Natl Acad Sci. 2014;111: 3002–3007. 10.1073/pnas.1319054111 24516146 PMC3939898

[31] Leslie AGW, Powell HR. Evolving Methods for Macromolecular Crystallography [Internet]. Evolving Methods for Macromolecular Crystallography. 2007 10.1007/978-1-4020-6316-9

[32] Kabsch W. XDS. Acta Crystallogr Sect D Biol Crystallogr. 2010;66: 125–132. 10.1107/S0907444909047337 20124692 PMC2815665

[33] Kabsch W. XDS. Acta Cryst (2010) D66, 125–132 [101107/S0907444909047337]. 2010; 1–8. 10.1107/S0907444909047337 20124692 PMC2815665

[34] Collaborative Computational Project N. Collaborative Computational Project, Number 4 (1994). The CCP4 Suite: programs for protein crystallography. Acta Crystallogr. 1994;D50: 760–763. 10.1107/S0907444994003112 15299374

[35] Karplus PA, Diederichs K. Assessing and maximizing data quality in macromolecular crystallography. Current Opinion in Structural Biology. 2015 pp. 60–68. 10.1016/j.sbi.2015.07.003 26209821 PMC4684713

[36] Yeates TO. Detecting and overcoming crystal twinning. Methods in Enzymology. 1997 pp. 344–358. 10.1016/S0076-6879(97)76068-3 9048378

[37] Murshudov GN, Skubák P, Lebedev AA, Pannu NS, Steiner RA, Nicholls RA, et al REFMAC5 for the refinement of macromolecular crystal structures. Acta Crystallogr Sect D Biol Crystallogr. 2011;67: 355–367. 10.1107/S0907444911001314 21460454 PMC3069751

[38] Emsley P, Cowtan K. Coot: Model-building tools for molecular graphics. Acta Crystallogr Sect D Biol Crystallogr. 2004;60: 2126–2132. 10.1107/S0907444904019158 15572765

[39] Delano WL, Bromberg S. PyMOL User’s Guide [Internet]. DeLano Scientific LLC. 2004.

[40] Jójárt B, Martinek TA. Performance of the general amber force field in modeling aqueous POPC membrane bilayers. J Comput Chem. 2007;28: 2051–2058. 10.1002/jcc.20748 17431937

[41] Tribello GA, Bonomi M, Branduardi D, Camilloni C, Bussi G. PLUMED 2: New feathers for an old bird. Comput Phys Commun. 2014;185: 604–613. 10.1016/j.cpc.2013.09.018

[42] Souaille M, Roux B. Extension to the weighted histogram analysis method: combining umbrella sampling with free energy calculations. Comput Phys Commun. 2001;135: 40–57. 10.1016/S0010-4655(00)00215-0

[43] Giorgino T, Laio A, Rodriguez A. METAGUI 3: A graphical user interface for choosing the collective variables in molecular dynamics simulations. Comput Phys Commun. 2017;217: 204–209. 10.1016/j.cpc.2017.04.009

[44] Becchetti a, Gamel K, Torre V. Cyclic nucleotide-gated channels. Pore topology studied through the accessibility of reporter cysteines. J Gen Physiol. 1999;114: 377–92. 10469728 10.1085/jgp.114.3.377 PMC2229457

[45] Mazzolini M, Punta M, Torre V. Movement of the C-helix during the gating of cyclic nucleotide-gated channels. Biophys J. 2002;83: 3283–3295. 10.1016/S0006-3495(02)75329-0 12496096 PMC1302404

[46] Kielland J. Individual Activity Coefficients of Ions in Aqueous Solutions. J Am Chem Soc. 1937;59: 1675–1678. 10.1021/ja01288a032

[47] Marchesi A, Mazzolini M, Torre V. Gating of cyclic nucleotide-gated channels is voltage dependent. Nat Commun. 2012;3 10.1038/ncomms1972 22828633

[48] Menini A. Currents carried by monovalent cations through cyclic GMP-activated channels in excised patches from salamander rods. J Physiol. 1990;424: 167–185. 10.1113/jphysiol.1990.sp018061 1697343 PMC1189807

[49] Sesti F, Eismann E, Kaupp UB, Nizzari M, Torre V. The multi-ion nature of the cGMP-gated channel from vertebrate rods. J Physiol. 1995;487: 17–36. 10.1113/jphysiol.1995.sp020858 7473247 PMC1156596

