# Supplementary figures and images for: The permeation mechanism of organic cations through a CNG mimic channel
Source: PLoS Comput Biol. 2018 Aug 2;14(8):e1006295. doi: 10.1371/journal.pcbi.1006295 (PMC6091977; doi:10.1371/journal.pcbi.1006295)

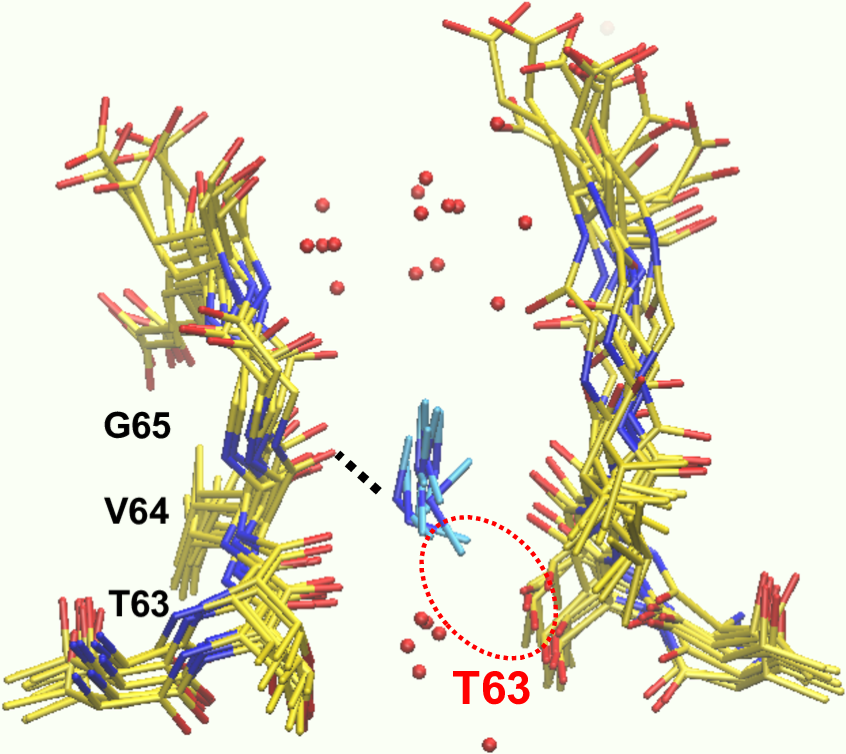

Supplement: S1 Fig — Superimposition of 10 instantaneous configurations taken from MD simulations showing the DMA coordination in S3c. The black dashed line refers to H-bonds, while the red dashed circle indicates the hydrophobic interactions. The average number of water coordinating DMA is about 1.5. For simplicity only the residues 62–66 are shown for two opposite subunits in the tetramer. DMA cation is shown as cyan sticks and well-ordered waters as red spheres. (TIF) [file pcbi.1006295.s001.tif]

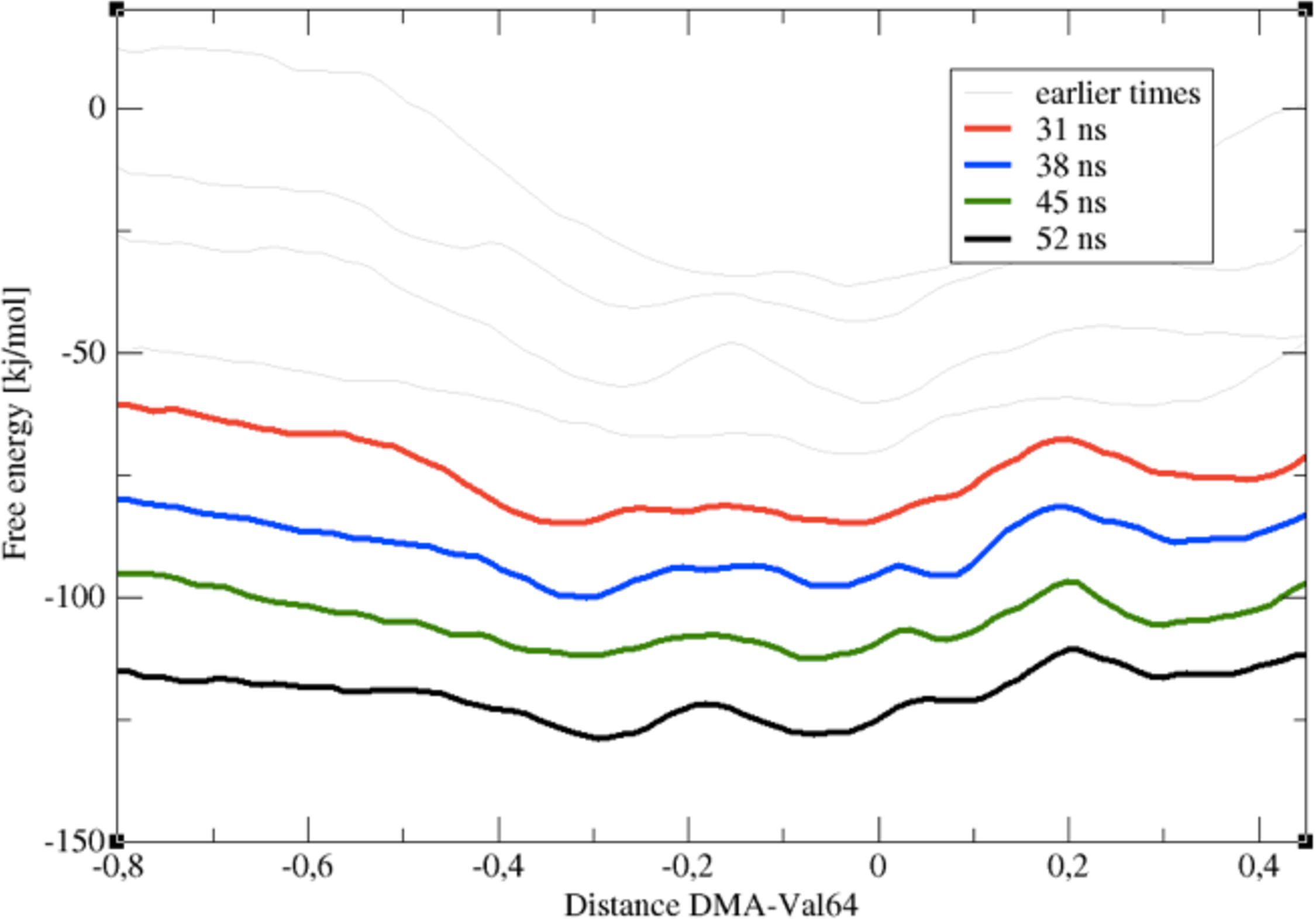

Supplement: S2 Fig — Free energy profiles (kj/mol) as a function of the distance of the DMA from its binding site, represented by center of mass of Val64 (nm). Different colors refer to different times. (TIF) [file pcbi.1006295.s002.tif]

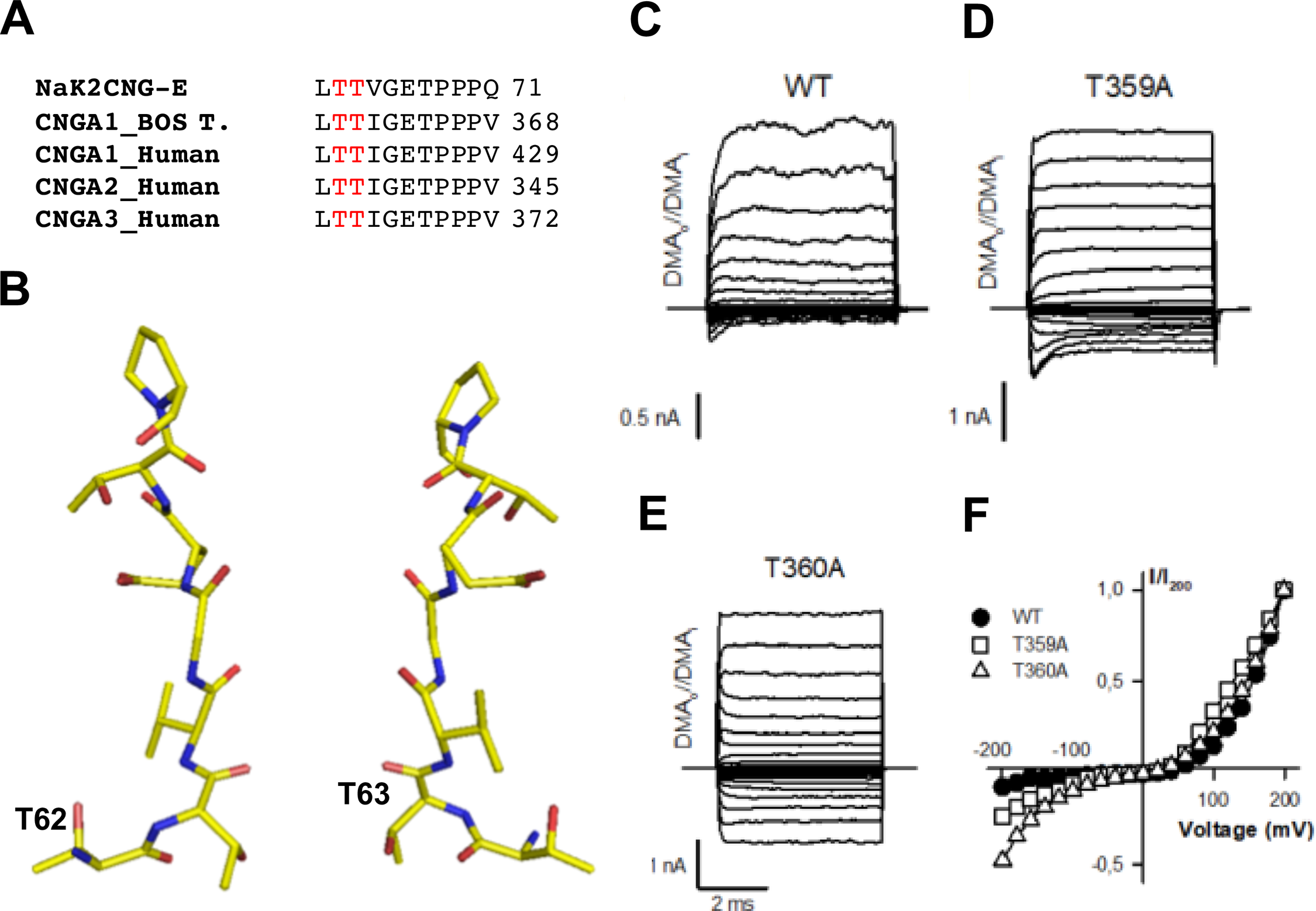

Supplement: S3 Fig — (A) Sequence alignment in the selectivity filter of the NaK2CNG-E, bCNGA1, hCNGA1, hCNGA2 and hCNGA3 channels. In red, the Thr which have been mutated in Ala. (B) Structure of the NaK2CNG-E selectivity filter (PDB ID: 4R7C) showing the position of the T62 and T63 residues. (C-E) Representative currents observed in the presence of symmetrical solutions (110 mM) of DMA for WT channel (C), T359A (D) and T360A (E) mutants. Voltage commands are from -200 to +200 mV in 20 mV steps (holding potential of 0 mV). (F) Normalized I–V relationship for the recordings in A-C (black dots, open squares, and open triangles refer to WT channel, T359A, and T360A mutants, respectively. (TIF) [file pcbi.1006295.s003.tif]

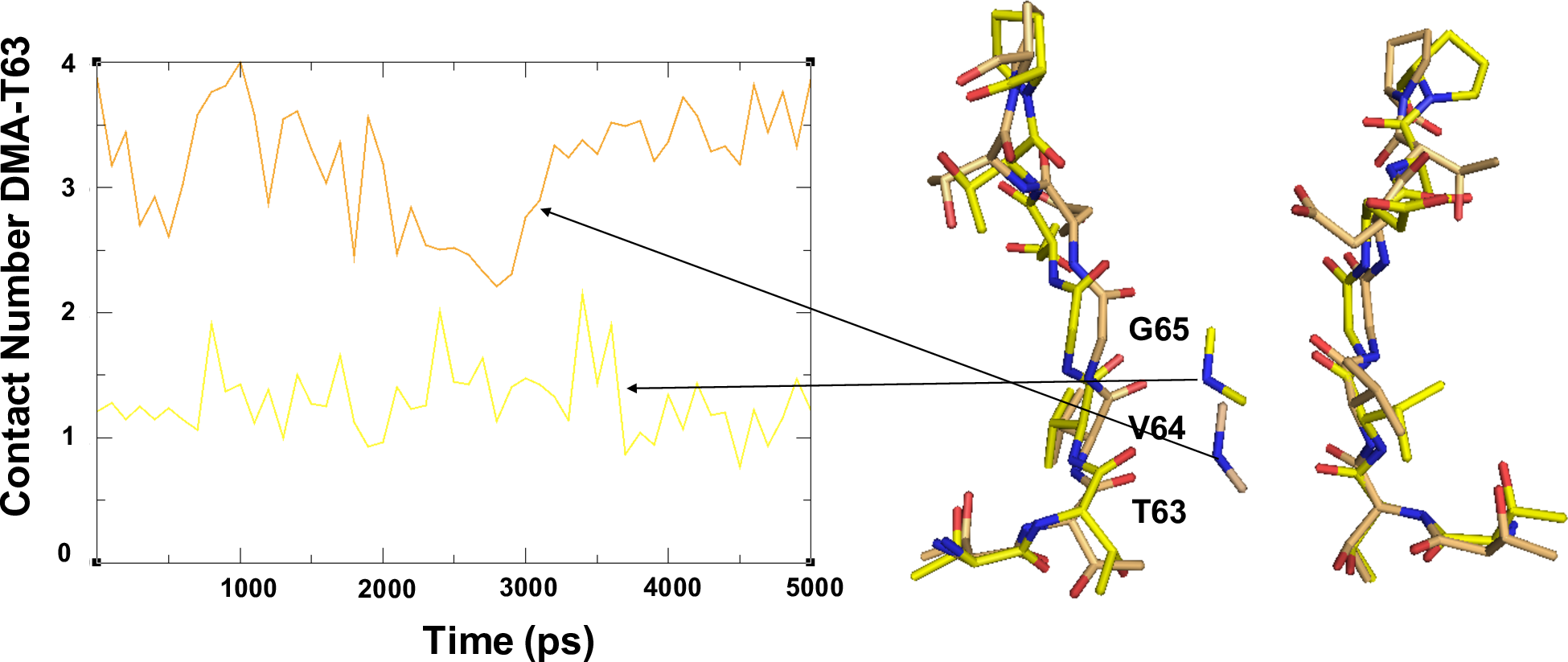

Supplement: S4 Fig — Right side: The tetrameric structure of a single DMA and 2 DMA complexes are superimposed and only the residues 62–68 are shown for the opposite subunits in the tetramer. The DMA complex is colored in yellow, while DMA-DMA complex in wheat. Changes in the position of the DMA can be observed. Left side: The contact numbers between the N of the DMA and the O of the hydroxyl group of T63 in both single DMA and DMA-DMA complex structures. (TIF) [file pcbi.1006295.s004.tif]

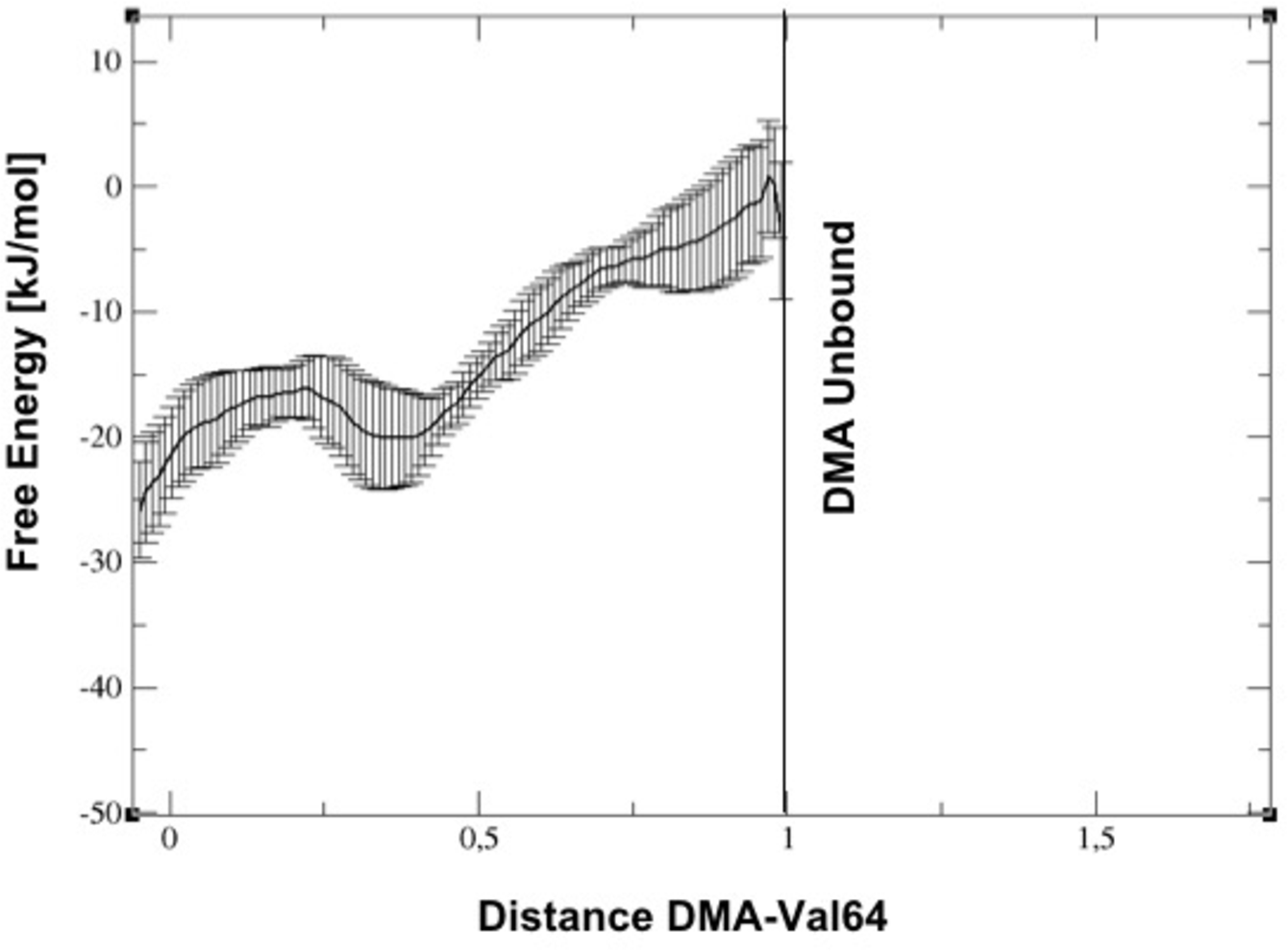

Supplement: S5 Fig — Free Energy profile (kJ/mol) of the DMA-DMA configuration along the distance of the DMA from its binding site, represented by center of mass of Val64 (nm). The free energy profile is shown with the relative error bars estimated by block analysis [29]. (TIF) [file pcbi.1006295.s005.tif]
