# Supplementary material for: The permeation mechanism of organic cations through a CNG mimic channel
Source: PLoS Comput Biol. 2018 Aug 2;14(8):e1006295. doi: 10.1371/journal.pcbi.1006295 (PMC6091977; doi:10.1371/journal.pcbi.1006295)
Supplement: S1 Appendix — A detailed explanation of the model reported in Fig 5. (PDF) [file pcbi.1006295.s006.pdf]

S1 Appendix

A model for DMA permeation through the CNGA1 channels. As discussed in the main text, we assume that backward crossings from the intracellular to the extracellular medium are not allowed. In this case, the flux J of ions X from the intracellular to the extracellular medium is

$$J = p_R k_R + p_D \widetilde{k_R}$$

(S1)

At the stationary state, the probabilities satisfy the following equations:

$$[X_{in}]k_L p_E + k^- p_R + \widetilde{k_R} p_D - k^+ p_L = 0$$

$$k^+ p_L - k^- p_R - [X_{in}]k_L p_R - k_R p_R = 0$$

$$[X_{in}]k_L p_R - \widetilde{k_R} p_D = 0$$

$$-[X_{in}]k_L p_E + k_R p_R = 0$$

(S2)

Here, k^+ is the transition rate from the binding site at the left to that at the right and k^- is the corresponding reverse rate. The rate $\widetilde{k_R}$ is associated to a transition between the state with double occupancies (D) and the state with a single ion occupying the left side (L). Notice that due to the interaction between the two ions $\widetilde{k_R}$ is likely to be significantly larger than k_R.

Ionic permeation through a strong and a weak binding site. The permeation of ion D through an energy profile composed by two barriers and one well with the corresponding rate constants k_i, as illustrated in Fig.3A, is governed by the equation:

$$d/dt(c_D) = D\, k_1(1-c_D) - c_D\,(k_{-1} + k_2)$$

(S3)

where c_D is the probability that the well is occupied by the permeation ion whose concentration is D. At the steady state the flux of ion D from the left to the right of the energy profile on Fig.3A is:

$$I_{out} = k_2 D/(K+D) \quad \text{with} \quad K= (k_{-1} + k_2)/k_1$$

(S4)

It is well known that if the two barriers B_1 and B_2 have a similar height K is simply related to the well depth W by the simple relation

$$K= \text{const} \exp (- W/RT)$$

(S5)

Therefore, if the energy profile of an ionic channel has two barriers with the same height and one single well, the depth of the well, i.e. the strength/affinity of the associated binding site, can be determined by electrophysiological measurements aiming to establish the dependence of the amplitude of the permeating current at a given voltage V as a function of the concentration D of the permeating ion.

The crystallographic data and the BE-META simulations (Fig.1 and Fig.3) both indicate the existence of two binding sites or wells, so that we have one deep and one more shallow wells. The equations corresponding to the permeation of an ion from left to right whose concentration is D is governed by the equations:

$$d/dt(c_1)= D\, k_1(1-c_1) - c_1 (k_{-1} + k_2) + k_{-2}\, c_2 (1-c_2)$$

(S6)

$$d/dt(c_2)= k_2\, c_1(1-c_2) - c_2 (k_{-2} + k_3)$$

(S7)

where c_1 and c_2 are the probability that the binding sites/wells 1 and 2 are occupied.

At the steady state the two equations (S6) and (S7) are both equal to 0 and after some algebra can be solved. The final net flux is given by

$$I_{out} = k_3\, X$$

(S8)

Where X is the solution of the second order equation

$$-k_2B/A\ x^2\ + x\ (\ k_2B/A\ \ -k_2Dk_1k_2/A\ –(k_{-2}+k_3))\ +\ k_2D\ k_1\ k_2/A\ =0$$

(S9)

$$\text{Where } A= Dk_1k_2 + k_{-1}\ k_2 + k_2^2 + k_2\ k_{-2} \quad \text{and} \quad B= k_2\ k_{-2} + k_{-2}^2 + k_{-2}\ k_3$$

(S10)

An investigation of the structure of the solution shows that by increasing the concentration D the flux I_{out} saturates, but that the concentration of D producing half of the maximal current is not related in a simple way to the depth of the well, as clearly as in the case of a single well (see Fig3A).

$$p_E + p_R + p_L + p_D = 1$$

(S11)

allow determining pE, pL, pR, pD, and then by some algebra we obtain the flux J:

$$J = \frac{[X_{in}]k_L k^+ \widetilde{k_R}([X_{in}]k_L + k_R)}{(k^+ + \widetilde{k_R})[X_{in}]2k_L^2 + \widetilde{k_R}\,(k_R + k^- + k^+)[X_{in}]k_L + k_R k^+ \widetilde{k_R}}$$

(S12)
