# Supplementary material for: The permeation mechanism of organic cations through a CNG mimic channel
Source: PLoS Comput Biol. 2018 Aug 2;14(8):e1006295. doi: 10.1371/journal.pcbi.1006295 (PMC6091977; doi:10.1371/journal.pcbi.1006295)
Supplement: S2 Appendix — Here are reported the Plumed files used to obtain the Free Energy profile reported in Fig 2. (PDF) [file pcbi.1006295.s007.pdf]

S2 Appendix

1. **Plumed file (-0.9, 0.5 corresponding to black color in Fig3A)**

c: COM ATOMS=738,2280,3822,5364

cv1: DISTANCE ATOMS=c,76306 COMPONENTS

cv2: DISTANCE ATOMS=5387,769

cv3: DISTANCE ATOMS=761,5395

cv4: DISTANCE ATOMS=2303,3853

cv5: DISTANCE ATOMS=3845,2311

cv6: DISTANCE ATOMS=754,5380

cv7: DISTANCE ATOMS=2296,3838

cv8: GYRATION TYPE=RADIUS ATOMS=754,2296,3838,5380

UPPER_WALLS ARG=cv1.z AT=**0.5** KAPPA=5000.0 EXP=2 EPS=1 OFFSET=0 LABEL=uwall_1

LOWER_WALLS ARG=cv1.z AT=**-0.9** KAPPA=5000.0 EXP=2 EPS=1 OFFSET=0 LABEL=lwall_1

UPPER_WALLS ARG=cv2 AT=1.3 KAPPA=5000.0 EXP=2 EPS=1 OFFSET=0 LABEL=uwall_2

LOWER_WALLS ARG=cv2 AT=0.5 KAPPA=5000.0 EXP=2 EPS=1 OFFSET=0 LABEL=lwall_2

UPPER_WALLS ARG=cv3 AT=1.3 KAPPA=5000.0 EXP=2 EPS=1 OFFSET=0 LABEL=uwall_3

LOWER_WALLS ARG=cv3 AT=0.5 KAPPA=5000.0 EXP=2 EPS=1 OFFSET=0 LABEL=lwall_3

UPPER_WALLS ARG=cv4 AT=1.3 KAPPA=5000.0 EXP=2 EPS=1 OFFSET=0 LABEL=uwall_4

LOWER_WALLS ARG=cv4 AT=0.5 KAPPA=5000.0 EXP=2 EPS=1 OFFSET=0 LABEL=lwall_4

UPPER_WALLS ARG=cv5 AT=1.3 KAPPA=5000.0 EXP=2 EPS=1 OFFSET=0 LABEL=uwall_5

LOWER_WALLS ARG=cv5 AT=0.5 KAPPA=5000.0 EXP=2 EPS=1 OFFSET=0 LABEL=lwall_5

UPPER_WALLS ARG=cv6 AT=1.4 KAPPA=5000.0 EXP=2 EPS=1 OFFSET=0 LABEL=uwall_6

LOWER_WALLS ARG=cv6 AT=0.4 KAPPA=5000.0 EXP=2 EPS=1 OFFSET=0 LABEL=lwall_6

UPPER_WALLS ARG=cv7 AT=1.2 KAPPA=5000.0 EXP=2 EPS=1 OFFSET=0 LABEL=uwall_7

LOWER_WALLS ARG=cv7 AT=0.4 KAPPA=5000.0 EXP=2 EPS=1 OFFSET=0 LABEL=lwall_7

UPPER_WALLS ARG=cv8 AT=0.55 KAPPA=5000.0 EXP=2 EPS=1 OFFSET=0 LABEL=uwall_8

LOWER_WALLS ARG=cv8 AT=0.25 KAPPA=5000.0 EXP=2 EPS=1 OFFSET=0 LABEL=lwall_8

2. Plumed file (0.35,1.2 corresponding to green color in Fig 3A)

c: COM ATOMS=738,2280,3822,5364

cv1: DISTANCE ATOMS=c,76306 COMPONENTS

cv2: DISTANCE ATOMS=5387,769

cv3: DISTANCE ATOMS=761,5395

cv4: DISTANCE ATOMS=2303,3853

cv5: DISTANCE ATOMS=3845,2311

cv6: DISTANCE ATOMS=754,5380

cv7: DISTANCE ATOMS=2296,3838

cv8: GYRATION TYPE=RADIUS ATOMS=754,2296,3838,5380

UPPER_WALLS ARG=cv1.z AT=**1.2** KAPPA=5000.0 EXP=2 EPS=1 OFFSET=0 LABEL=uwall_1

LOWER_WALLS ARG=cv1.z AT=**0.35** KAPPA=5000.0 EXP=2 EPS=1 OFFSET=0 LABEL=lwall_1

UPPER_WALLS ARG=cv2 AT=1.3 KAPPA=5000.0 EXP=2 EPS=1 OFFSET=0 LABEL=uwall_2

LOWER_WALLS ARG=cv2 AT=0.5 KAPPA=5000.0 EXP=2 EPS=1 OFFSET=0 LABEL=lwall_2

UPPER_WALLS ARG=cv3 AT=1.3 KAPPA=5000.0 EXP=2 EPS=1 OFFSET=0 LABEL=uwall_3

LOWER_WALLS ARG=cv3 AT=0.5 KAPPA=5000.0 EXP=2 EPS=1 OFFSET=0 LABEL=lwall_3

UPPER_WALLS ARG=cv4 AT=1.3 KAPPA=5000.0 EXP=2 EPS=1 OFFSET=0 LABEL=uwall_4

LOWER_WALLS ARG=cv4 AT=0.5 KAPPA=5000.0 EXP=2 EPS=1 OFFSET=0 LABEL=lwall_4

UPPER_WALLS ARG=cv5 AT=1.3 KAPPA=5000.0 EXP=2 EPS=1 OFFSET=0 LABEL=uwall_5

LOWER_WALLS ARG=cv5 AT=0.5 KAPPA=5000.0 EXP=2 EPS=1 OFFSET=0 LABEL=lwall_5

UPPER_WALLS ARG=cv6 AT=1.4 KAPPA=5000.0 EXP=2 EPS=1 OFFSET=0 LABEL=uwall_6

LOWER_WALLS ARG=cv6 AT=0.4 KAPPA=5000.0 EXP=2 EPS=1 OFFSET=0 LABEL=lwall_6

UPPER_WALLS ARG=cv7 AT=1.2 KAPPA=5000.0 EXP=2 EPS=1 OFFSET=0 LABEL=uwall_7

LOWER_WALLS ARG=cv7 AT=0.4 KAPPA=5000.0 EXP=2 EPS=1 OFFSET=0 LABEL=lwall_7

UPPER_WALLS ARG=cv8 AT=0.55 KAPPA=5000.0 EXP=2 EPS=1 OFFSET=0 LABEL=uwall_8

LOWER_WALLS ARG=cv8 AT=0.25 KAPPA=5000.0 EXP=2 EPS=1 OFFSET=0 LABEL=lwall_8

3. Plumed file (0.9, 1.6 corresponding to blue color in Fig.3A)

c: COM ATOMS=738,2280,3822,5364

cv1: DISTANCE ATOMS=c,76306 COMPONENTS

cv2: DISTANCE ATOMS=5387,769

cv3: DISTANCE ATOMS=761,5395

cv4: DISTANCE ATOMS=2303,3853

cv5: DISTANCE ATOMS=3845,2311

cv6: DISTANCE ATOMS=798,5424

cv7: DISTANCE ATOMS=2340,3882

cv8: GYRATION TYPE=RADIUS ATOMS=798,2340,3882,5424

UPPER_WALLS ARG=cv1.z AT=**1.6** KAPPA=5000.0 EXP=2 EPS=1 OFFSET=0 LABEL=uwall_1

LOWER_WALLS ARG=cv1.z AT=**0.9** KAPPA=5000.0 EXP=2 EPS=1 OFFSET=0 LABEL=lwall_1

UPPER_WALLS ARG=cv2 AT=1.3 KAPPA=5000.0 EXP=2 EPS=1 OFFSET=0 LABEL=uwall_2

LOWER_WALLS ARG=cv2 AT=0.5 KAPPA=5000.0 EXP=2 EPS=1 OFFSET=0 LABEL=lwall_2

UPPER_WALLS ARG=cv3 AT=1.3 KAPPA=5000.0 EXP=2 EPS=1 OFFSET=0 LABEL=uwall_3

LOWER_WALLS ARG=cv3 AT=0.5 KAPPA=5000.0 EXP=2 EPS=1 OFFSET=0 LABEL=lwall_3

UPPER_WALLS ARG=cv4 AT=1.3 KAPPA=5000.0 EXP=2 EPS=1 OFFSET=0 LABEL=uwall_4

LOWER_WALLS ARG=cv4 AT=0.5 KAPPA=5000.0 EXP=2 EPS=1 OFFSET=0 LABEL=lwall_4

UPPER_WALLS ARG=cv5 AT=1.3 KAPPA=5000.0 EXP=2 EPS=1 OFFSET=0 LABEL=uwall_5

LOWER_WALLS ARG=cv5 AT=0.5 KAPPA=5000.0 EXP=2 EPS=1 OFFSET=0 LABEL=lwall_5

UPPER_WALLS ARG=cv6 AT=1.4 KAPPA=5000.0 EXP=2 EPS=1 OFFSET=0 LABEL=uwall_6

LOWER_WALLS ARG=cv6 AT=0.4 KAPPA=5000.0 EXP=2 EPS=1 OFFSET=0 LABEL=lwall_6

UPPER_WALLS ARG=cv7 AT=1.4 KAPPA=5000.0 EXP=2 EPS=1 OFFSET=0 LABEL=uwall_7

LOWER_WALLS ARG=cv7 AT=0.4 KAPPA=5000.0 EXP=2 EPS=1 OFFSET=0 LABEL=lwall_7

UPPER_WALLS ARG=cv8 AT=0.95 KAPPA=5000.0 EXP=2 EPS=1 OFFSET=0 LABEL=uwall_8

LOWER_WALLS ARG=cv8 AT=0.6 KAPPA=5000.0 EXP=2 EPS=1 OFFSET=0 LABEL=lwall_8

4. Plumed file (1.4, 2.0 corresponding to violet color in Fig. 3A)

c: COM ATOMS=738,2280,3822,5364

cv1: DISTANCE ATOMS=c,76306 COMPONENTS

cv2: DISTANCE ATOMS=798,5424

cv3: DISTANCE ATOMS=2340,3882

cv4: GYRATION TYPE=RADIUS ATOMS=798,2340,3882,5424

UPPER_WALLS ARG=cv1.z AT=**2.0** KAPPA=5000.0 EXP=2 EPS=1 OFFSET=0 LABEL=uwall_1

LOWER_WALLS ARG=cv1.z AT=**1.4** KAPPA=5000.0 EXP=2 EPS=1 OFFSET=0 LABEL=lwall_1

UPPER_WALLS ARG=cv2 AT=1.4 KAPPA=5000.0 EXP=2 EPS=1 OFFSET=0 LABEL=uwall_2

LOWER_WALLS ARG=cv2 AT=0.4 KAPPA=5000.0 EXP=2 EPS=1 OFFSET=0 LABEL=lwall_2

UPPER_WALLS ARG=cv3 AT=1.4 KAPPA=5000.0 EXP=2 EPS=1 OFFSET=0 LABEL=uwall_3

LOWER_WALLS ARG=cv3 AT=0.4 KAPPA=5000.0 EXP=2 EPS=1 OFFSET=0 LABEL=lwall_3

UPPER_WALLS ARG=cv4 AT=0.95 KAPPA=5000.0 EXP=2 EPS=1 OFFSET=0 LABEL=uwall_4

LOWER_WALLS ARG=cv4 AT=0.6 KAPPA=5000.0 EXP=2 EPS=1 OFFSET=0 LABEL=lwall_4
